# First Record of *Caloapenesia* (Hymenoptera, Bethylidae) from China with Description of One New Species †

**DOI:** 10.3390/insects16050451

**Published:** 2025-04-24

**Authors:** Chunhong Wang, Junhua He, Celso O. Azevedo, Xuexin Chen

**Affiliations:** 1 Ministry of Agriculture and Rural Affairs Key Laboratory of Molecular Biology of Crop Pathogens and Insects, Zhejiang University, Hangzhou 310058, China; joycek0324@163.com; 2 Institute of Insect Sciences, College of Agriculture and Biotechnology, Zhejiang University, Hangzhou 310058, China; 3 Departamento de Ciências Biológicas, Universidade Federal do Espírito Santo, Av. Fernando Ferrari 514, Goiabeiras, Vitória 29075-910, ES, Brazil; bethylidae@gmail.com; 4 State Key Laboratory of Rice Biology and Breeding, Zhejiang University, Hangzhou 310058, China; 5 Zhejiang Provincial Key Laboratory of Biology and Ecological Regulation of Crop Pathogens and Insects, Zhejiang University, Hangzhou 310058, China

**Keywords:** Pristocerinae, geographic distribution, new records, key for males

## Abstract

The genus *Caloapenesia*, only known from the Oriental region, comprises nineteen species, seventeen of which are recorded from Thailand and the Philippines, countries adjacent to China. During our examination of bethylid specimens from southern China, we discovered *Caloapenesia xui* sp. nov. from Guangdong Province, representing the northernmost record for the genus. The new species is described and illustrated, and an updated key to the males of *Caloapenesia* is provided.

## Simple Summary

The Oriental genus *Caloapenesia* (Hymenoptera: Bethylidae), belonging to Pristocerinae, is reported from China for the first time. The new species, *Caloapenesia xui* sp. nov., is described based on morphological comparisons. An updated key to all species of *Caloapenesia* is provided. The diagnostic characters used for species identification and the geographic distribution of this genus are discussed.

## 1. Introduction

The Pristocerinae genus *Caloapenesia* was established by Terayama (1995) based on the type species *Caloapenesia thailandiana* Terayama [1]. Initially, the genus was defined based only on the males due to the lack of female specimens. Azevedo (2004) [2] expanded the genus by adding one species, *Caloapenesia brevis*, and described the apterous female of this genus. In his study, the palpal formula of the female is 5:3, which is different to the male’s 6:3 [2]. Gobbi and Azevedo (2014) reassessed the generic characters of *Caloapenesia* using specimens from Indonesia, the Philippines, and Thailand, and corrected the male palpal formula to 5:3 from 6:3, as described in a previous study [3]. They also described sixteen new species of this genus and provided a key to all known *Caloapenesia* species.

To date, the genus includes nineteen species, exhibiting high species diversity in countries adjacent to China: twelve species recorded in Thailand and five in Vietnam [2,3]. During our study of Chinese bethylids, we identified a new species, *Caloapenesia xui* sp. nov., from Guangdong Province, representing the northernmost record for the genus, but still in the Oriental region. In this study, we describe and illustrate this new species and provide a revised key to the species of *Caloapenesia* worldwide.

## 2. Materials and Methods

### 2.1. Specimens

The specimens examined in this study were collected through sweeping net and Malaise traps. All the specimens examined in this study are deposited in the Parasitic Hymenoptera Collection of Zhejiang University, Hangzhou, China (ZJUH).

### 2.2. Preparation for Morphological Study

Male genitalia were dissected using an apical curved micro insect pin; subsequent cleaning of the hypopygium and genitalia was performed according to the method of Martinelli et al. [4]. The dissected hypopygium and the genitalia were stored in a microtube with glycerin. Morphological terminology follows Lanes et al. (2020) [5] and Brito et al. (2022) [6].

A Nikon stereomicroscope (SMZ800N, Nikon Corporation, Tokyo, Japan) was used for observation. Biometric measurements and photos of the external and the genitalia characters were taken through the digital microscope Keyence (VHX-X1, Keyence Corporation, Osaka, Japan). The photos were partly enhanced and laid out on a plate using Adobe Photoshop 2023.

## 3. Results

### 3.1. Taxomomy

Genus *Caloapenesia* Terayama, 1995

*Caloapenesia* Terayama, 1995: 882. Type species: *Caloapenesia thailandiana* Terayama, 1995, by original designation. Azevedo, 2004: 143–144; Gobbi and Azevedo, 2014: 506; Azevedo et al., 2018: 69–71.

**Diagnosis.** MALE. Mandible with five apical teeth. Median clypeal lobe clearly defined. Frons densely punctuated. Eye usually prominent, with long erect setae. Dorsal pronotal area long. Mesoscutum with notaulus and parapsidal furrow present. Fore wing with pterostigma absent, C vein obscure. Hypopygium with spiculum long and anterolateral apodeme reduced or absent, posterior margin slightly to strongly trilobate. Genitalia with harpe deeply divided into separate arms, dorsal harpe straight, slightly sclerotized and glabrous. FEMALE. Mandible with four apical teeth. Palpal formula 5:3. Clypeus with median lobe trapezoidal, median clypeal carina divided in two apically. Eye with numerous facets. Mesopleuron large in dorsal view, projected anteriorly. Metapectal-propodeal complex strongly constricted anteriorly. Mesotibia strongly spinose.

**Host.** Unknown.

**Distribution.** Oriental region.

**Remarks.** This genus was recorded from China for the first time.

### 3.2. Description of New Species

**Figure 1:**
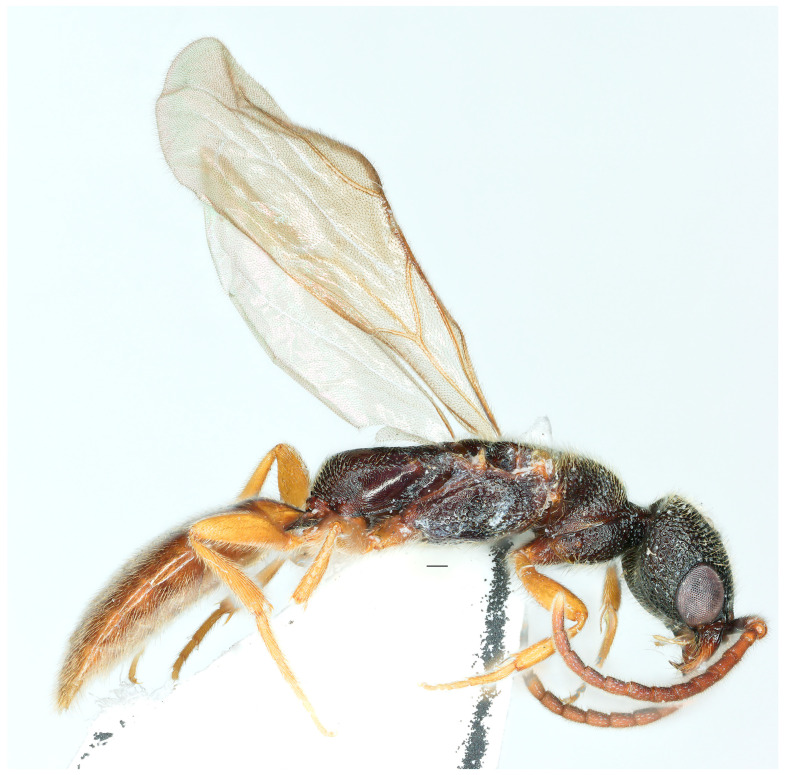
*Caloapenesia xui* Wang, He and Chen sp. nov., holotype, male. Habitus, lateral view. Scale bars: 0.15 mm.

**Figure 2:**
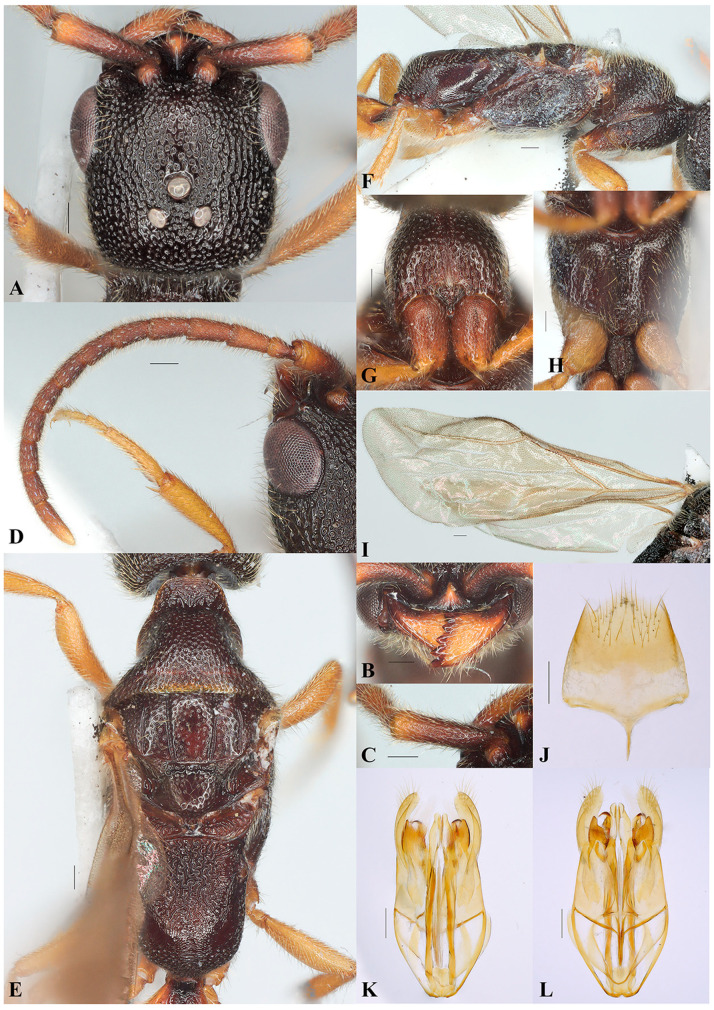
*Caloapenesia xui* Wang, He and Chen sp. nov., holotype, male. (**A**) Head, antero-dorsal view; (**B**) mandible; (**C**) scape; (**D**) antenna; (**E**) mesosoma, dorsal view; (**F**) mesosoma, lateral view; (**G**) propleuron and prosternum, ventral view; (**H**) mesopectum, ventral view; (**I**) fore wing; (**J**) hypopygium; (**K**) genitalia, dorsal view; and (**L**) genitalia, ventral view. Scale bars: 0.15 mm.

*Caloapenesia xui* Wang, He and Chen sp. nov. (Figure 1 and Figure 2)

**Diagnosis.** The new species *Caloapenesia xui* sp. nov. was included in the genus *Caloapenesia* for the median clypeal lobe well defined, dorsal pronotal area with anterior and lateral margin distinct (Figure 2E), eye with short setae, harpe of male genitalia bilobate, and pterostigma absent.

This new species is similar to the species *Caloapenesia heira* Gobbi and Azevedo, 2014 by having ocelli small, pedicel short, and head longer than wide. However, the new species can be distinguished from the latter by having the median clypeal lobe subtrapezoidal and with yellow patch medially, the sides of head posterior to eyes nearly parallel, the occipital carina visible in dorsal view, the pronotal flange castaneous, the median area of mesoscutum with punctures nearly as dense as pronotum, the median area of mesoscutellum punctate, the dorsal surface of metapectal-propodeal disc rugose with posterior one-third rugulose, the lateral margin of hypopygium straight, the ventral harpe broader than dorsal harpe, whereas *C. heira* has the median clypeal lobe subtrapezoidal incurved and wholly black, sides of head behind eyes converging posterad, the occipital carina not visible in dorsal view, the pronotal flange yellowish brown, the median area of mesoscutum with punctures sparser than pronotum, the median area of mesoscutellum not punctate, the dorsal surface of metapectal-propodeal disc with basal half rugose, the lateral margin of hypopygium outcurved, the ventral harpe as wide as dorsal one.

**Description.** Holotype. Male (Figure 1). Body length 6.08 mm. Fore wing length 3.99 mm.

Colour. Dark castaneous to black, metasoma yellowish brown. Mandible yellow, castaneous basally, apical teeth castaneous. Median clypeal lobe with yellow patch medially. Antenna castaneous, scape and flagellomere XI yellow apically. Legs yellow, procoxa light castaneous. Fore wing hyaline, tinged with light castaneous; veins light castaneous to castaneous.

Head (Figure 2A). Head 1.12 × longer than wide. Mandible with five distal teeth (Figure 2B). Median clypeal lobe subtrapezoidal, anterior margin with small median tooth; medial clypeal carina not exceeding antennal foramen. Antenna with suberect pubescence, nearly half as long as width of flagellomere; first four antennomeres in ratio about 5:1:2:2 (Figure 2B,D); pedicel 1.04 × as long as wide; flagellomere I 1.74 × as long as wide; flagellomere IX 1.61 × as long as wide. Antennal torulus prominent. Frons coriaceous with dense punctures; WF 0.6 × WH. Eye with erect pubescence, WF 1.69 × LE. Vertex coriaceous with dense punctures, profile of temples nearly parallel in antero-dorsal view; anterior ocellus slightly posterior to supra-ocular line; DAO 0.16 × WF. POL 1.36 × AOL, OOL 1.09 × WOT; vertex crest nearly straight; occipital carina visible in dorsal view. DPV 1.94 × DAO. Gena coriaceous with dense punctures; medioccipito-genal line carinate.

Mesosoma (Figure 2E–H). Dorsal pronotal area coriaceous with dense punctures, median length including cervical pronotal area 0.48 × width along posterior pronotal margin; cervical pronotal area long, visible in dorsal view, irregularly rugose; cervical pronotal area and lateral pronotal area coriaceous with dense punctures. Mesoscutum coriaceous with dense punctures; width of mesoscutum between tegulae 1.87 × its median length; median mesonotal sulcus absent; notauli complete and punctate, converging posterad; parapsidal signum complete, weakly punctate; median area of mesoscutum with sparser punctures than pronotum. Mesoscutellum shiny with dense punctures; mesoscuto-scutellar sulcus straight and foveolate. Metascutellum weakly coriaceous; metanotal trough with dense oblique carinae. Metapectal-propodeal disc distinctly rugose, with posterior one-third rugulose; lateral marginal and transverse posterior carina absent; propodeal declivity distinctly foveolate; anterior half of lateral surface of metapectal-propodeal complex weakly coriaceous, and posterior half rugulose. Propleuron weakly coriaceous with dense punctures. Prosternum depressed, posterior margin with median projection (Figure 2G). Mesopleuron weakly coriaceous with dense punctures; posterior oblique sulcus of mesopleuron foveolate (Figure 2F); ventral surface of mesopectus coriaceous with shallow punctures; subpleural signum present; mesodiscrimen developed and foveolate (Figure 2H). Metasternal plate rugose (Figure 2H).

Wings (Figure 2I). Costal cell of fore wing long, 2r-rs&Rs vein rises basal to costal cell. Hind wing with five distal hamuli.

Metasoma. Weakly coriaceous with tiny punctures; metasomal tergum I depressed medially and rugulose. Posterior margin of hypopygium trilobate, median lobe distinctly longer than lateral one; lateral margin straight and converging posterad; spiculum present; anterolateral apodeme present, inconspicuous (Figure 2J).

Genitalia (Figure 2K,L). Harpe bilobate, ventral harpe with long setae apically and broader than dorsal harpe; dorsal harpe glabrous; inner margin of digitus with short setae in ventral view; aedeagus uniformly wide.

Female. Unknown.

**Materials examined.** Holotype, ♂, China: Guangdong, Shaoguan, Dinghu Mountain, Zaifu Xu, 11.VIII–12.VIII.2005 (No. 2000609390).

**Distribution.** China (Guangdong).

**Etymology.** This species is named in memory of Professor Zaifu Xu (South China Agricultural University) for his contribution to the taxonomic study of bethylids from China.

Key to males (modified from Gobbi and Azevedo, 2014)

1. Pedicel more than 0.75 × flagellomere I..........................................................................2
− Pedicel less than 0.6 × flagellomere I..............................................................................8
2. Flagellomeres short, less than 0.35 × scape....................................................................3
− Flagellomeres median-sized, more than 0.35 × scape...................................................4
3. Metapectal-propodeal disc without metapostnotal-propodeal carina; hypopygeal median lobe convex; hypopygeal lateral lobe angulate; anterolateral apodeme spiniform......................................................................*C. inyara*
− Metapectal-propodeal disc with conspicuous metapostnotal-propodeal carina; hypopygeal median lobe subangulate; hypopygeal lateral lobe outcurved and very short; anterolateral apodeme rectangular...............................................*C. rikawa*
4. Ocelli small, less than 0.18 × WF.....................................................................................5
− Ocelli large, more than 0.23 × WF...................................................................................7
5. Metapectal-propodeal disc with conspicuous metapostnotal-propodeal carina...........................................................................................................................................6
− Metapectal-propodeal disc with metapostnotal-propodeal carina inconspicuous...................................................................................................................................*C. lani*
6. Hypopygium strongly trilobate with median lobe rounded..............................*C. diba*
− Hypopygium slightly trilobate with median lobe angulate..........................*C. jailuna*
7. Median clypeal lobe rounded; occipital carina not visible in dorsal view; head black; mesosoma dark castaneous...................................................................*C. leptata*
− Median clypeal lobe angulate; occipital carina visible in dorsal view; head and mesosoma testaceous.............................................................................*C. philippinensis*
8. Ocelli small, less than 0.18 × WF....................................................................................9
− Ocelli large, more than 0.23 × WF................................................................................18
9. Median clypeal lobe trapezoidal..................................................................................10
− Median clypeal lobe rounded or subrectangular.......................................................11
10. Corners of median clypeal lobe angulate; occipital carina complete and visible in dorsal view; hypopygium strongly trilobate, without anterolateral apodeme.............................................................................................................*C. launeci*
− Corners of median clypeal lobe rounded; occipital carina ill defined; hypopygium slightly trilobate, with anterolateral apodeme rectangular......*C. edas*
11. Median clypeal lobe subrectangular..........................................................................12
− Median clypeal lobe rounded......................................................................................13
12. Flagellomere I less than 1.5 × as long as wide; occipital carina not visible in dorsal view; metapectal-propodeal disc with metapostnotal-propodeal carina conspicuous.......................................................................................................*C. mugra*
− Flagellomere I more than 2.0 × as long as wide; occipital carina visible in dorsal view; metapectal-propodeal disc without metapostnotal-propodeal carina........................................................................................................................................*C. nadaili*
13. Pedicel distinctly short, 0.27 × flagellomere I; dorsalmost tooth of mandible sharp.....................................................................................................................*C. brevis*
− Pedicel nearly 0.6 × flagellomere I; dorsalmost tooth rounded or blunt.............14
14. Head longer than wide, LH at least 1.10 × WH..........................................................15
− Head as long as wide.....................................................................................................16
15. Sides of head posterior to eyes converging posterad; median clypeal lobe without yellow patch; median area of mesoscutellum polished, not punctate; lateral margin of hypopygium outcurved; dorsal harpe as wide as ventral harpe.......................................................................................................................*C. heira*
− Sides of head posterior to eyes nearly parallel; median clypeal lobe with yellow patch medially; median area of mesoscutellum punctate; lateral margin of hypopygium straight; dorsal harpe narrower than ventral harpe................................................................................................................................*C. xui* sp. nov.
16. Occipital carina without ventral projection................................................................17
− Occipital carina with ventral projection.........................................................*C. paruwa*
17. Hypopygeal posterior margin slightly trilobate; anterolateral apodeme absent..............................................................................................................................*C. arbeni*
− Hypopygeal posterior margin strongly trilobate; anterolateral apodeme conspicuous and spiniform...................................................................................*C. ana*
18. Eye median-sized, WF 1.15–1.35 × HE.........................................................................19
− Eye very large, WF less than 1.10 × HE..............................................................*C. supra*
19. Hypopygeal posterior margin strongly trilobate...................................*C. thailandiana*
− Hypopygeal posterior margin straight with median lobe angulate........*C. sabeli*

## 4. Discussion

Gobbi and Azevedo’s (2014) [3] revision of *Caloapenesia* examined an extensive suite of morphological characters, and the morphometric ratios were integrated into the dichotomous key for the species identification of this genus. However, relying solely on morphometric ratios for species identification may lead to misclassification, as these ratios depending on precise linear measurements are susceptible to specimen preparation artefacts. For example, in Couplet 2 of their key, the distinction between “flagellomeres short: less than 0.35 × scape length” and “flagellomeres median-sized: more than 0.35 × scape length” could be affected by the mounting angle, potentially leading to inconsistent identification results. Furthermore, there are no molecular data available for reliable species identification within *Caloapenesia*, as all available molecular data about this genus have been generated for the phylogenetic studies without species-level identification. For example, Alencar et al. (2018) reported two 28S rRNA sequences [7], and Santos et al. (2024) generated four UCE (ultraconserved element) records [8], all with species unidentified. Therefore, integrating geometric morphometrics, molecular data, and broader sampling will be essential for future studies of this genus to resolve species boundaries robustly.

Additionally, the high diversity of this genus in the Oriental region, coupled with the recent discovery of *Caloapenesia xui* sp. nov. in China, underscores the need for further sampling in adjacent regions (e.g., southern China, India, Myanmar, and the Malay Peninsula). Such efforts could uncover additional species and provide deeper insights into this genus’s biogeographic history, particularly its northern limits.

## Data Availability

The original contributions presented in the study are included in the article, further inquiries can be directed to the corresponding author.

## Abbreviations

The following abbreviations are used for morphological terms and biometric measurements:

AOL	width between anterior and posterior ocellus, measured as minimum length in frontal view
LH	length of head, measured in antero-dorsal view, from apex of clypeus to vertex
OOL	shortest distance from a posterior ocellus to nearest eye margin
WF	width of frons, measured in antero-dorsal view, its minimum width
WH	width of frons, measured in antero-dorsal view, its minimum width
WOT	width of ocellar triangle, measured in antero-dorsal view, maximum width including ocelli
DPV	distance between posterior ocelli and vertex crest in antero-dorsal view
